# Diagnostic polymorphisms in the mitochondrial cytochrome b gene allow discrimination between cattle, sheep, goat, roe buck and deer by PCR-RFLP

**DOI:** 10.1186/1471-2156-5-30

**Published:** 2004-10-05

**Authors:** Ina Pfeiffer, Joachim Burger, Bertram Brenig

**Affiliations:** 1 Institute of Veterinary Medicine, Georg-August-University of Göttingen, Groner Landstrasse 2, D-37073 Göttingen, Germany; 2 Institute of Anthropology, Johannes Gutenberg-University of Mainz, Colonel Kleinmann Weg 2, D-55099 Mainz, Germany

## Abstract

**Background:**

As an alternative to direct DNA sequencing of PCR products, random PCR-RFLP is an efficient technique to discriminate between species. The PCR-RFLP-method is an inexpensive tool in forensic science, even if the template is degraded or contains only traces of DNA from various species.

**Results:**

Interspecies-specific DNA sequence polymorphisms in the mitochondrial cytochrome b gene were analyzed using PCR-RFLP technology to determine the source (i.e., species) of blood traces obtained from a leaf.

**Conclusions:**

The method presented can be used for the discrimination of cattle (*Bos taurus*), sheep (*Ovis aries*), goat (*Capra hircus*), roe buck (*Capreolus capreolus*) and red deer (*Cervus elaphus*).

## Background

Determination of the species from which traces of source material, such as blood stains on a leaf, originate can sometimes be a difficult task in forensic DNA analysis. For instance, insurance claims that involve car accidents with animals require authentication. Species identification is also essential in food quality control-procedures or for the detection and identification of animal material in food samples. Numerous analytical methods that rely on protein analysis have been developed for species identification, such as electrophoresis techniques [1,2], immunoassays [3] and liquid chromatography [4]. However, proteins are heat labile and lose their biological activity. Furthermore, their presence and characteristics depend on special cell types. Thus, for species identification, DNA analysis would be preferred over protein analysis. The first genetic approach for determination of species identity was the dot-blot technique [5]. At present, polymerase chain reaction (PCR) is the technique of choice for species identification [6]. Some PCR approaches are RAPD-PCR (random amplified polymorphic DNA fingerprints) [7] and others are focused on RFLP analysis [8]. In this work we present a more sensitive method to detect DNA from degraded samples. Usually, the required specimen (hair, a part of skin or a piece of meat) contains degraded DNA and the PCR products must be cloned before sequencing [9]. Existing techniques consist of laborious and costly DNA sequencing procedures. We therefore used a less time-consuming PCR method to amplify a short mitochondrial (mt) DNA fragment. The PCR-RFLP allowed discrimination between different species even in cases in which the source material contained only degraded DNA. The mitochondrial cytochrome b gene has been used in phylogenetic as well as in forensic investigations [10-12] and has been shown in a variety of studies to be a very useful DNA-region for species determination [13-18]

However, before applicable for routine analysis, species-specific diagnostic polymorphisms or mutations must be determined.

If these mutations affect restriction enzyme sites, a simple PCR-RFLP can be used as a "sceening tool" for detection.

One area in which the PCR-RFLP screening tool would be useful is in training hunting hounds. Before hounds are accepted and approved for hunting, they have to be evaluated by different tests. These tests include an examination of the dog's ability for winding and trailing. To examine these abilities normally a track is prepared consisting of minute amounts of blood from game animals, e. g., wild boar, spotted on a few leaves that are distributed in the forest or field. A trained hound should be capable of winding and trailing without any problems. However, in a case that came to our attention, hounds were unable to wind and it was assumed by the owners that the examiner had used blood from domestic animals. Hence, the question as to the source of the track (i.e., blood from domestic or game animals) was open.

To solve this problem we established genetic test (PCR-RFLP) that focused on the use of diagnostic polymorphisms in the mitochondrial cytochrome b gene: Our method can be used for the discrimination between the following mammal species: cattle (*Bos taurus*), sheep (*Ovis aries*), goat (*Capra hircus*), roe buck (*Capreolus capreolus*) and red deer (*Cervus elaphus*).

## Results and discussion

**Figure 1:**
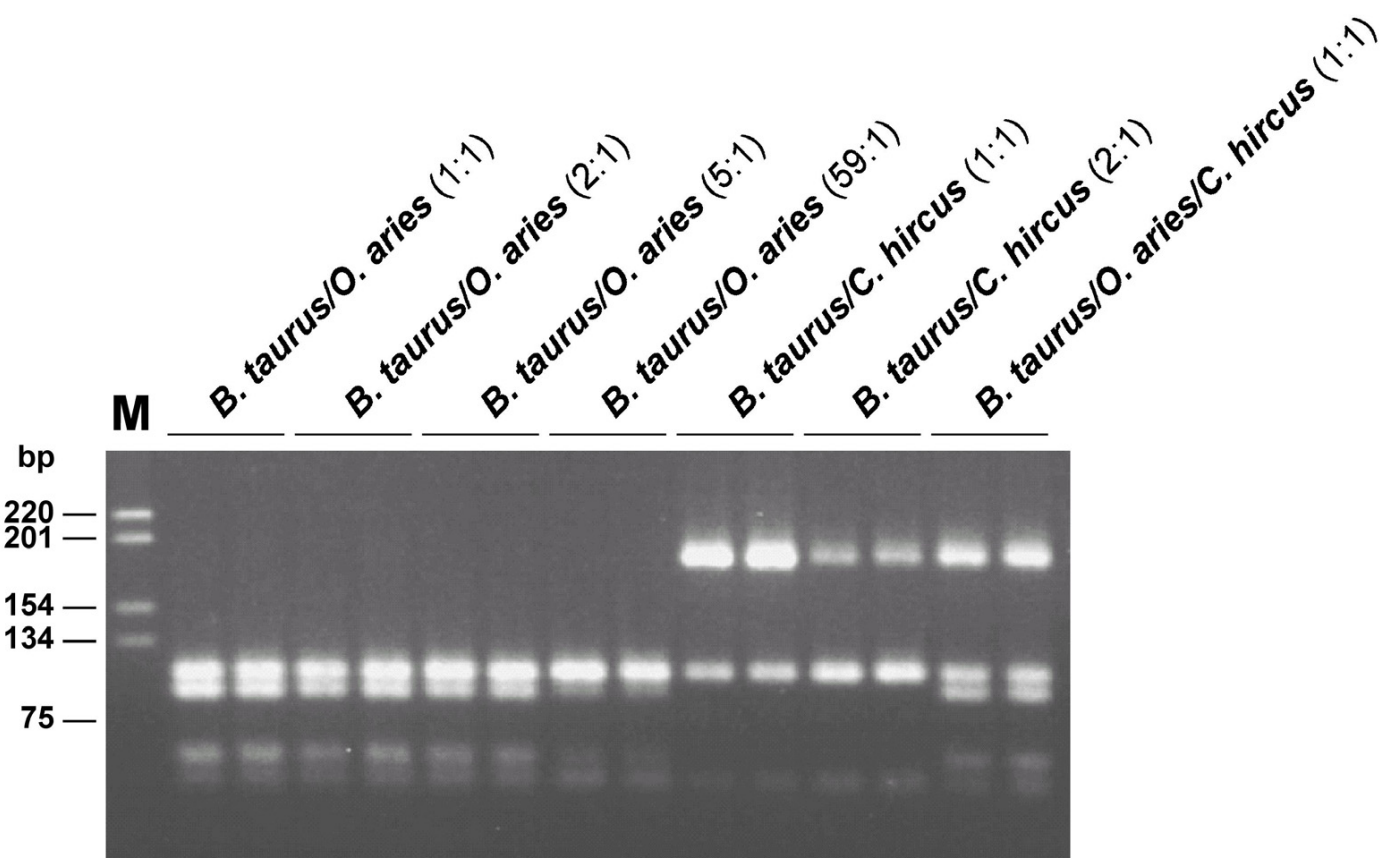
Restriction cleavage patterns of DNA mixtures with varying amounts of target DNA.

For the analysis, DNA was prepared from blood remains on different leaves. The DNA was amplified as described in Methods. Figure 1 shows RFLP results of five different species (*Bos taurus*, *Ovis aries*, *Capra hircus*, *Capreolus capreolus *and *Cervus elaphus*) and the pattern of bands from the blood sample of one of the dry leaves. As controls, DNA-samples from known species were used. Specific RFLP patterns that distinguish between 5 species are analyzed here. To estimate the exact size of fragments, DNA sequence information was used. *C. hircus *shows a single fragment of 182 bp, whereas with *C. capreolus *a fragment of 162 bp was obtained. The RFLP pattern of *C. elaphus *consisted of 2 fragments of 108 bp and 54 bp respectively. The blood sample on the leaf shows four fragments: 114 bp and 68 bp-fragments, which are characteristic for *B. taurus, *and a 105 bp and 75 bp fragment which can be assigned to *O. aries. *This result indicates that the blood sample on the leaf represents a mixture from two species (*B. taurus *and *O. aries)*. The results were obtained from two independent DNA extractions and confirmed several times by independent PCRs, as well as by DNA sequencing. Amplifications were repeated and species identification was verified by diagnostic Dloop sequencing [19]. The Dloop DNA sequence of the PCR amplified fragment of the blood track was aligned with the mtDNA sequence of *B. taurus *and three nucleotide differences were observed.

In addition to species determination using PCR-RFLP analysis, we tested for different DNA ratios of mixed samples, by which an assignment to a species is still possible. A valid assignment of a mixture of *B. taurus *and *O. aries *is still possible for a ratio of 59:1(Fig. 1).

The data clearly illustrate that it is possible to identify the species from unknown material using PCR-RFLP, provided that comparison to a known species is performed on the same gel.

Furthermore, the PCR-RFLP enables the observation of frequent contaminants such as cattle DNA in routine diagnostic labs. While direct sequencing of coamplified endogenous DNA would lead to multiple sequences, the PCR-RFLP is able to separate two signals. Sometimes, possible contaminants (such as human DNA) can lead to false results, but with the designed primer pair this problem was circumvented. For this reason we designed maximally discriminatory primers and the mismatches in each primer are sufficient to exclude human DNA under stringent PCR conditions. The primers were tested with different amounts of added DNA. This method can be used for analysis of mixed samples, since up to three species in different proportions can be determined. The RFLP test using other tissues, e.g., muscle, hair with roots, bones, and saliva, yielded reproducible results. Faeces have not been tested so far.

However, when minimizing target DNA, the bands tend to fade away on the agarose gel. The presence of a specific PCR-RFLP for the species analysed, a fragment length of less than 200 bp and the exclusion of contaminating sequences improved methods for existing species determination. The principle of RFLP is often used in food analysis [20,21]. However, the new PCR-RFLP method is capable of analysing degraded DNA, especially in forensic cases. Furthermore, the PCR-RFLP utilizes only a small fraction of apomorphic sites.

The speed and the efficiency of current nucleotide technology, such as automatic sequencing, will permit the identification of additional taxa. However, the establishment of a PCR-RFLP test would likely need further extensive experimentation.

## Conclusions

In summary, we were able to show that a simple PCR-RFLP is efficient in differentiating between *B. taurus*, *O. aries*, *C. hircus*, *C. capreolus *and *C. elaphus*. However, after further development, the tested mitochondrial nucleotide sequences may allow the forensic identification of other animal species.

## Methods

### DNA extraction

#### DNA extraction (Normal samples)

DNA was extracted from blood and tissue samples using QIAamp^® ^Tissue Kit (QIAGEN GmbH, Hilden, Germany) according to the manufacturers' handbook. Isolated DNA was diluted in 50 μL HPLC-H_2_O and used for further analyses.

#### DNA extraction (Trace material e.g. bones)

Bone samples were roughly ground with a pestle and mortar, then finely powdered in a Retsch mill. Bone powder (0.3 g) was incubated in 1.5 mL of 0.5 M EDTA (pH 8.3) for 20 h while rotating. The suspension was centrifuged for 4 min at 4000 rpm. The supernatant was transferred to a fresh tube or to an automated nucleic acids extraction system (Nucleic Acid Extractor 341A, Applied Biosystems) and 1.6 mL sterile distilled water (Ampuwa, Fresenius) was added. As the extraction procedure was automated the volumes of reagents dispensed may have varied between runs. Five hundred microliters of Proteinase K was added and the mixture incubated for 1 h at 58°C with shaking. Three milliliters of phenol/chloroform/isoamyl alcohol (25:24:1, pH 7.5–8.0) were added and the mixture was further incubated at room temperature for 6 min while shaking. The phases were allowed to separate by incubating at room temperature for 8 min without shaking and the organic phase and interphase, if present, were discarded. Chloroform (4.5 mL, 100%) was added to the aqueous phase and the mixture incubated for 6 min at room temperature while shaking. The phases were again allowed to separate by incubating at room temperature for 8 min without shaking and the organic phase and interphase, if present, were discarded. Ninety microliters of sodium acetate (pH 4.5) and 3.2 mL of 100% isopropanol were added followed by incubation for 2 min with shaking. Five microliters of Glasmilk (Dianova) were added and the suspension was shaken for another 10 min. To obtain a pellet, the solution was filtered through Precipitette filters (Applied Biosystems) or centrifuged for 3 min at 5000 rpm. The pellet was washed with 80% ethanol and eluted into 50 μL sterile distilled water (Ampuwa, Fresenius). Five to ten microliters of extract were used for PCR amplification or the extract was stored at -20°C. Glasmilk was not removed prior to amplification.

### PCR-RFLP

For RFLP analysis, a 195 bp long PCR fragment was amplified from mitochondrial cytochrome b region. The following primer pair was used for amplification, CB7u (5'-GCGTACGCAATCTTACGATCAA-3') and CB7l (5'-CTGGCCTCCAATTCATGTGAG-3').

The PCR was carried out in a total volume of 50 μL consisting of 10 ng DNA, 60 mM KCl; 12 mM Tris-HCl; 2.5 mM MgCl_2_; 150 μM dNTPs; 0,18 μM of each Primer and 2 U AmpliTaq Gold (PE Applied Biosystems). Cycling conditions included a denaturation step at 95°C for 3 minutes, followed by 32 cycles of 95°C for 1 minute, primer annealing at 54°C for 1 minute and elongation at 72°C for 1 minute in a thermocycler (Hybaid).

The 195 bp fragment was digested with TSP509 (New England Biolabs) for two hours at 65°C. The resulting fragments were separated by gelelectrophoresis in a 2.5 % agarose gel.

### MtDNA Dloop PCR sequencing reaction

A single fragment of the mitochondrial DNA Dloop region was amplified using primers Dloopu (5'-AAATGTAAAACGACGACGGCCAGTAATCCCAATAACTCAACAC-3') and Dloopll (5'-AAACAGGAAACAGCTATGACCACTCATCTAGGCATTTTC-3').

Amplifications were performed in a final volume of 20 μL in 10 × PCR buffer (15 mM MgCl_2_, pH 8.3) and Q-solution, 100 μM for each dNTP, with 1 M Taq Polymerase and 10 pmol of each primer. Four microlitres of the DNA-extract were added to the PCR mix. The amplification was carried out with initial denaturation at 95°C for 10 min, followed by 35 cycles of one denaturation step at 94°C for 40 sec, primer annealing at 52°C for 40 sec and primer extension at 72°C for 45 sec in a Hybaid thermocycler. PCR-products were purified using the QIAEX II Gel Extraction Kit (QIAGEN GmbH, Hilden, Germany) according to the manufacturers' instructions. Sequencing was performed using ABI-Prism™ Dye Kit V3 (Applied Biosystems) in a 10 μL volume containing 2 μL purified PCR-product and 5 pmol of primer. Sequencing reactions underwent 27 cycles of 30 sec at 94°C, 30 sec at 50°C and 3 min at 60°C in a Techne thermocycler. The dye terminators were removed by sephadex-G45 column purification (Millipore). Sequencing reactions were electrophoresed for 2 h on an ABI Prism^® ^3100 genetic analyzer (Applied Biosystems) according to the manufacturers' instructions.

### Sample selection

In order to test the specifity of the technique the following numbers of specimens were tested:

7 unrelated samples from cattle: Holstein Frisian, Charolais, Limousin, Angus

7 unrelated samples from sheep

7 unrelated samples from goat

7 unrelated samples from deer collected from Lower Saxony and North Hesse

7 unrelated samples from roe deer collected from Lower Saxony

Regarding closely related species, we analyzed mouflon DNA (*Ovis aries musimon*) and observed approximately 100% sequence identity compared with *Ovis aries*. Furthermore, we investigated different cattle breeds and we could not find any sequence differences within the tested cytochrome b DNA-fragment.

## Authors' contributions

IP performed the DNA extractions, PCR-RFLP analysis and mtDNA Dloop DNA sequencing.

JB developed and provided the PCR-RFLP protocol for species identification.

BB was responsible for funding, supervision of the research project, manuscript writing and editing as well as scientific correspondence.

**Figure 2:**
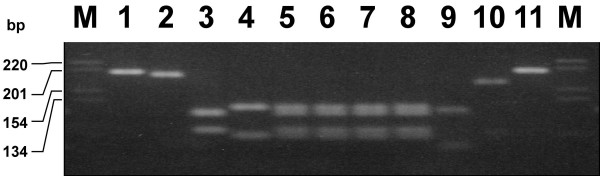
Restriction profiles of the 195 bp cytochrome b PCR fragments showing interspecies-specific polymorphism between *B. taurus*, *O. aries*, *C. hircus*, *C. capreolus *and *C. elaphus*. The unknown blood sample shows the fragment length of *B. taurus *and *O. aries*. Nr. 1 and Nr.11 controls *(Bos taurus*, undigested PCR product), Nr. 2: *C. hircus*., Nr. 3: *O. aries*, Nr. 4: *B. taurus*, Nr. 5, 6 and 7 blood sample on a leaf, Nr. 8: DNA mixture *B. taurus *and *O. aries*, Nr. 9: *C. elaphus*, Nr. 10: *C. capreolus*.
